# Implementation of a Successful Enhanced Recovery after Surgery Program in a Community Hospital

**DOI:** 10.7759/cureus.6029

**Published:** 2019-10-30

**Authors:** William Norcross, Timothy E Miller, Samuel Huang, Jay Kim, Skip Maza, Eddie Sanders, Colleen McCarthy, Earl Ransom

**Affiliations:** 1 Anesthesiology, Duke University School of Medicine, Durham, USA; 2 Anesthesia, Duke University School of Medicine, Durham, USA; 3 Urology, Duke Regional Hospital, Durham, USA; 4 Performance Services, Duke University School of Medicine, Durham, USA; 5 Statistician, Duke University School of Medicine, Durham, USA

**Keywords:** enhanced recovery programs (erps), enhanced recovery after surgery

## Abstract

Background

Enhanced recovery after surgery (ERAS) protocols have been shown to be effective at accelerating return to functioning, reducing length of stay, and reducing cost per encounter at major medical centers and health systems across the United States and Europe. Implementation in the community hospital setting has been considered more challenging due to a wide range of factors. This study demonstrates the successful creation of such a program in a community hospital in central North Carolina.

Methods

Starting in the spring of 2016, an anesthesiology-led, multidisciplinary ERAS team was formed with the purpose of developing an enhanced recovery after major urologic surgery program. A clinical protocol was developed by the team that met quarterly to review metrics. Outcome data were collected by chart review and compared to pre-ERAS values in a retrospective, nonrandomized, consecutive fashion and underwent statistical analysis.

Results

Overall, a reduction in both average and median length of stay (37% reduction) was observed in the post-ERAS group along with a reduction in 90-day readmission. Statistical analysis confirmed a very strong likelihood (p<.0001) that the ERAS protocol resulted in the observed reduction in the length of stay.

Discussion

This study demonstrated the feasibility of starting an ERAS program in a community hospital as well as the critical role that anesthesiology leadership can provide. An anesthesiology-led ERAS program offers a solution to some of the challenges faced by community hospitals regarding variable and silo-based care. ERAS pathways aim to implement standardized and coordinated evidence-based care protocols through multidisciplinary teams representing the entirety of the surgical encounter, leading to more consistent and favorable outcomes for patients and hospitals. This model can be applied to many other services in addition to the major urology effort described here.

## Introduction

Recent surgical quality improvement has been attributable to many factors, including the use of standardized perioperative care processes such as enhanced recovery after surgery (ERAS). ERAS protocols have been shown to reduce cost per encounter in major medical centers and health systems across the US and Europe [1-4]. The implementation of such standardized improvement processes, however, has been perceived to be challenging in the setting of smaller community hospitals. In these settings, provider and staff perceptions regarding practice autonomy, as well as limited resources, are unique burdens. We provide this case example of the development and maintenance of enhanced recovery protocols in a community hospital setting to provide further evidence of added value for community-based anesthesia groups in preparation for the transition toward value-based care models.

Part of this article is based on Norcross WP, Huang S, Kim J, Miller T, Sanders E, Maza S, McCarthy C, Ransom E; Implementation of a successful ERAS program in a community hospital; 2019.

## Materials and methods

In the Spring of 2016, an anesthesia-led, multidisciplinary ERAS team was formed to create an enhanced recovery after major urologic surgery program. The team consisted of stakeholders from the director of perioperative services. At the time, care for urology surgical patients involved several individual surgeon preferences and minimal care coordination. The team reviewed past institutional clinical experiences and developed a comprehensive perioperative clinical pathway, which was embraced by all key stakeholders in the urologic surgery program. The clinical pathway consisted of pre, intra, and postoperative interventions.

Preop phase of care

 1. Patient education materials distributed from the pre-anesthesia testing clinic, including the importance of smoking and alcohol cessation

 2. Preoptimization and risk stratification through perioperativist evaluation and testing

3. Protocol-driven routine pre-anesthesia testing

4. Preop hydration with two servings of complex carbohydrate drinks, one before bed and one two hours before arrival

Intraop phase of care

1. Pre-induction transverse abdominis plane (TAP) block with liposomal bupivacaine

2. Opiate-sparing anesthetic focused on rapid emergence

3. Multimodal postoperative nausea and vomiting (PONV) mitigation

4. Goal-directed fluid therapy (GDFT) utilizing a non-invasive cardiac output (NICO) monitor

5. Lung protective ventilation

6. Minimization of tubes, lines, and catheters

Postop phase of care

 1. Ambulation postoperative day (POD) #0 goal of 100 feet, mobilization at a minimum, 100 feet TID thereafter, frequency of vital signs was reduced to q4 to free up staff for ambulation

2. Clear liquid diet POD #0, advance to regular diet POD #1

3. Multimodal opiate sparing analgesia, avoidance of patient-controlled analgesia (PCA)

4. Multimodal PONV treatment

5. Anticipated discharge to home POD #1 for prostatectomy, POD #2 for nephrectomy

6. Direct contact information to the primary surgeon or representative to minimize the need for emergency department (ED) visits

Patients were collected in a non-randomized, consecutive fashion, with no outliers removed. A retrospective review of patients in the pre-ERAS baseline cohort was limited to a two-year window out of concern that collecting data further back in time would introduce bias due to changes in surgical staff. No other confounding factors were controlled for, including age, body mass index (BMI), comorbidities, or perioperative complications.

This program initially began as a quality improvement project. After six months, it became evident that the ERAS protocol was having a positive effect and the decision was made to collect pre-ERAS control data to compare to post-ERAS implementation. The control group was selected by retrospectively collecting data via an electronic medical record (EMR; Epic Systems, Madison, WI, US) chart review. Institutional review board (IRB) exemption was obtained in preparation for the analysis and presentation of results. Retrospective data via chart review between July 2016 and May 2018 for all patients undergoing major urologic surgical procedures (open retropubic prostatectomy, robotic prostatectomy, open nephrectomy, and laparoscopic-assisted nephrectomy) at Duke Regional Hospital were evaluated (Table 1).

**Table 1:** Effects of ERAS Pathway on Average Length of Stay by Specific Procedure ERAS: enhanced recovery after surgery (ERAS); ALOS: average length of stay

Procedure	Pre- ERAS ALOS	N	Post- ERAS ALOS	N	SD
Robotic Prostatectomy	5.38 days	14	1.91 days	55	3.98
Open Prostatectomy	4.22 days	27	1.93 days	32	1.31
Laparoscopic Nephrectomy	5.11 days	9	2.90 days	13	2.25
Open Nephrectomy	4.63 days	12	3.74 days	29	1.56

In this retrospective pre to post-ERAS implementation comparative analysis, the anesthetic in the pre-ERAS group was driven by anesthesia provider preference, with no standardized analgesia or fluid management protocols. Postoperative pain management relied primarily on intravenous opioids with inconsistently applied multimodal agents based on provider preference. Ambulation and diet advancement were at the discretion of the surgeon or the availability of nursing staff. In the post-ERAS group, providers followed an agreed clinical pathway that standardized pre-hydration and carbohydrate loading, a specialized anesthetic protocol including postoperative nausea and vomiting (PONV) prevention, goal-directed fluid therapy (GDFT), and opioid-sparing analgesia with liposomal bupivacaine transversus abdominis plane (TAP) block, and, finally, early and aggressive mobilization and diet advancement. In addition, patients were given specific instructions and information about the ERAS program to give patients a clear set of expectations when arriving for surgery. Patients were not consented to be part of the ERAS program.

Data collection was performed by hospital-based performance services. In the post-implementation of ERAS, quarterly dashboards tracked average and median length of stay index (Vizient, Inc., Texas, US), 30 and 90-day readmission, emergency department (ED) visits, and incidence of ileus.

The Wilcoxon- Breslow analysis was used for statistical analysis due to the non-normal nature of the length of stay data, values of less than zero being impossible.

## Results

**Figure 1:**
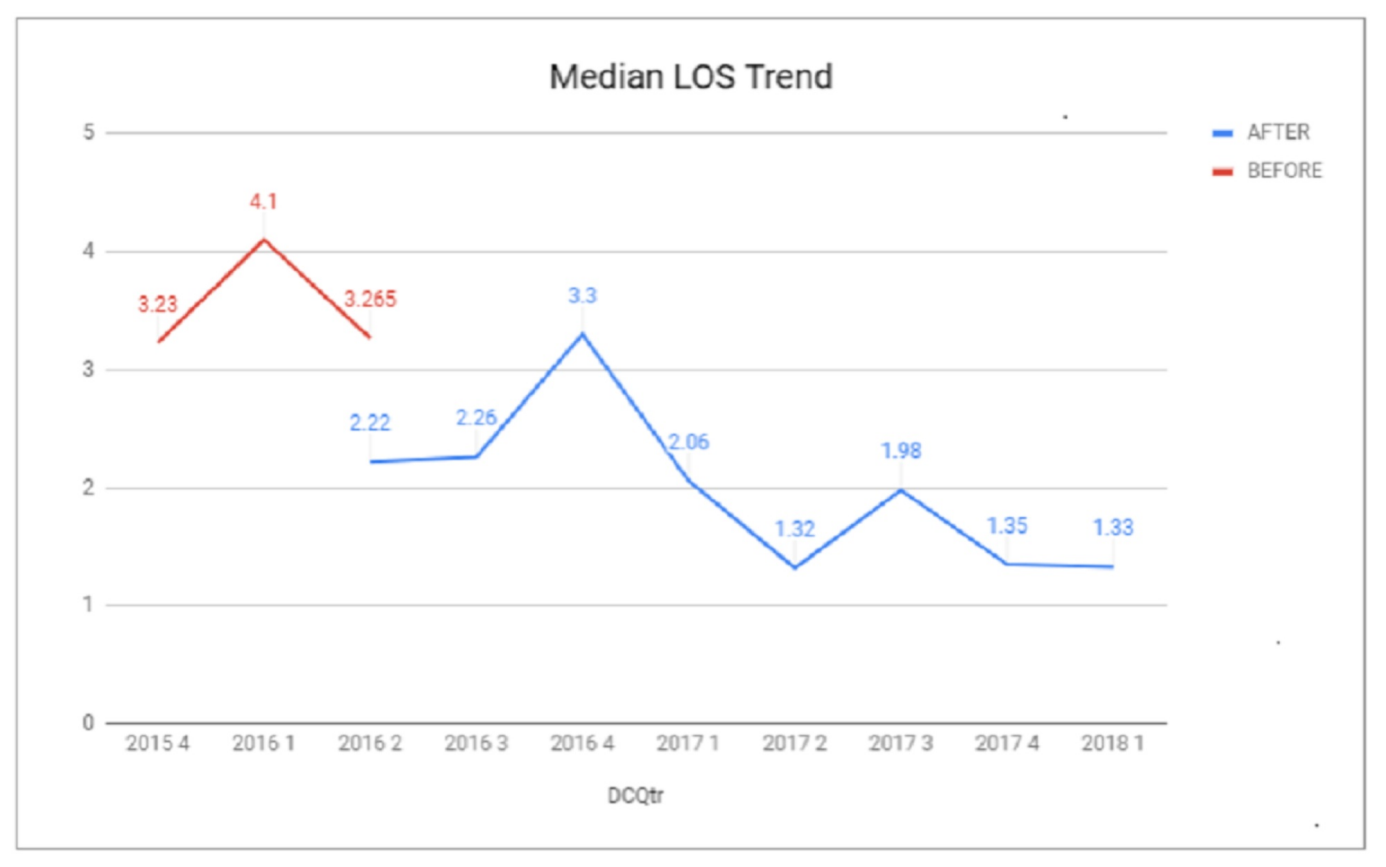
Median Length of Stay LOS: length of stay

**Figure 2:**
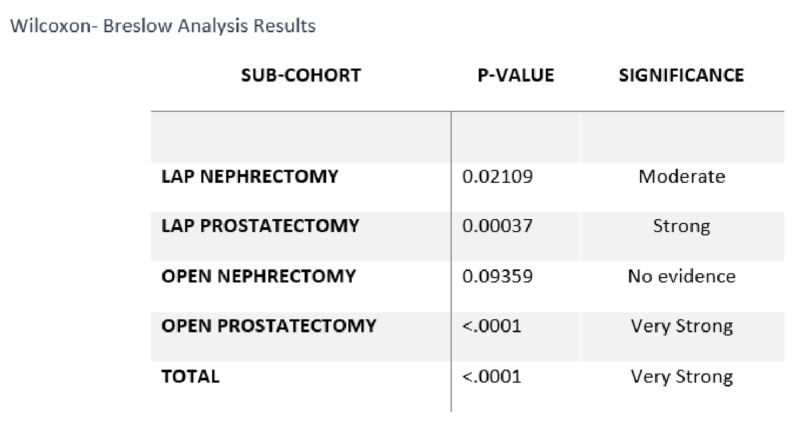
Statistical Analysis

**Figure 3:**
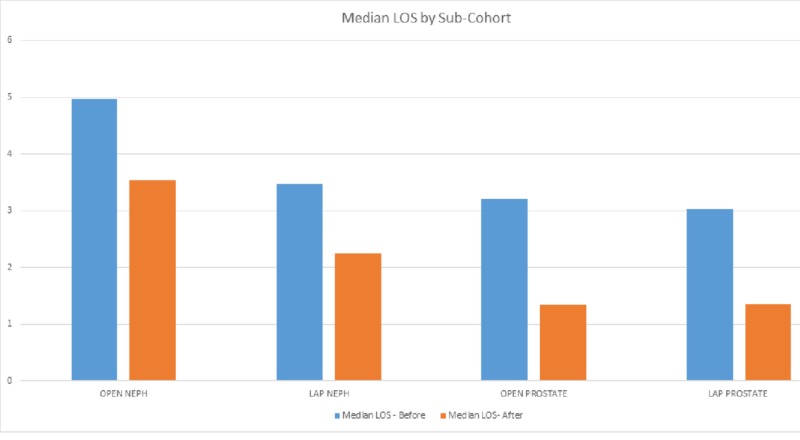
Median LOS by Sub-Cohort LOS: length of stay

**Figure 4:**
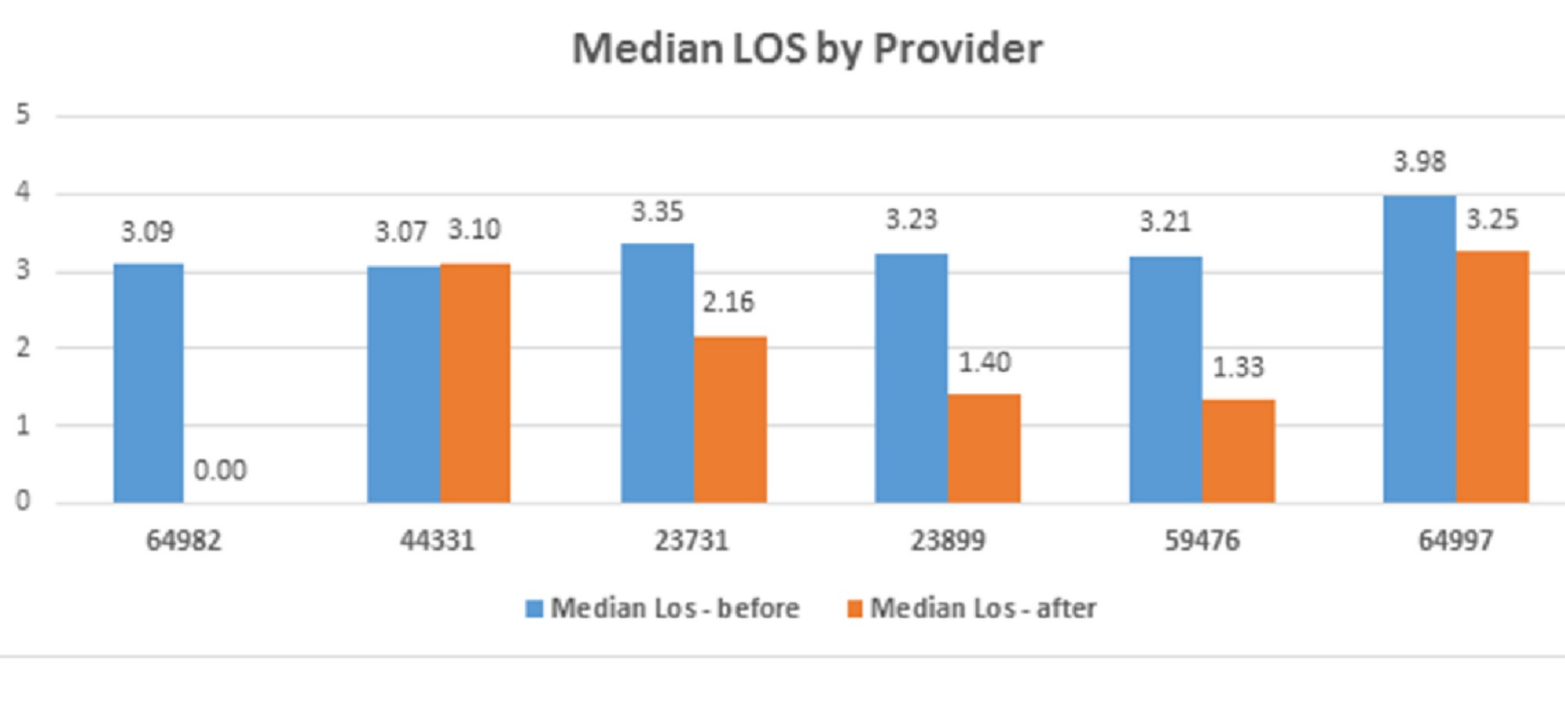
Median LOS by Provider LOS: length of stay

A chart review of pre-ERAS patients was compared to a post-ERAS cohort who underwent major urologic surgery by multiple surgeons between July 1, 2016, and May 1, 2018. Of the six surgeons represented in the baseline cohort, all were represented in the post-ERAS cohort. One surgeon retired early in the post-ERAS period; otherwise, there was no significant difference between surgeons. Sixty-two pre-ERAS patients were compared to a post-ERAS cohort of 129 patients. An overall reduction in both average and median LOS was observed between the post-ERAS cohort and the pre-ERAS cohort, with a reduction of median LOS from 3.24 days pre-ERAS (N=62) to 2.02 days post-ERAS (N=129) (37.75%, p=0001). A reduction in LOS was seen in specific surgical procedures that were evaluated (Figures 1-4). During the study period, a 90-day readmission rate of 3.1% was observed (three patients), a reduction from 4.84% pre-ERAS. The rate of patients returning to the ED within seven days fell from 6.45% pre-ERAS to 3.88% post-ERAS. Due to the low numbers of these variables, statistical analysis was not attempted.

## Discussion

We report on the successful implementation of an enhanced recovery after major urologic surgery program at a 365-bed community hospital in central North Carolina. ERAS programs offer solutions to some of the challenges faced in the community hospital setting, where processes may be fractionated and exist in silos of care. As well, suboptimal coordination of the perioperative encounter may exist among unaffiliated groups of physicians within the medical staff and among staff in different phases of care. For example, it has been demonstrated previously that without GDFT algorithms, the greatest indicator of the intraoperative fluid volume administered is the anesthesia provider; introducing unwanted variability that is correlated with a higher incidence of perioperative complications and prolonged length of hospital stay [5]. In addition, anesthesia care, surgical care, and nursing care may be poorly coordinated with limited feedback between groups.

ERAS pathways aim to implement standardized and coordinated evidence-based care protocols through multidisciplinary teams representing the entirety of the surgical encounter. Standard ERAS clinical protocols are aimed at preoperative medical optimization, perioperative opioid minimization, side-effect mitigation [6], and early ambulation and diet advancement. In addition, there is an emphasis on patient and staff education and empowerment, such that the patient and staff are active participants in the care and have realistic expectations of the perioperative experience. Regional anesthesia is frequently utilized as an essential part of opioid-sparing analgesia, as appropriate to the surgical procedure. In addition, GDFT [3,7-9] and lung-protective ventilation [10] intraoperative protocols help minimize variability in the management of fluids and mechanical ventilation. Representation from the staff at each phase of care in the development and implementation of care pathways spanning the continuum of care is essential for the proper coordination of care. Lastly, data collection and review is crucial in order to assess compliance with and the success of the program in meeting set goals. Through this process, the ERAS major urology program described here showed a significant and sustained reduction in LOS over an 18-month period of time, without an increased incidence of readmissions or complications.

Of note, the results reveal the individual surgeon had a significant impact on the effectiveness of the ERAS pathway. Specifically, one surgeon (44331) was more reluctant than others in aggressively discharging patients when analgesia, mobilization, and bowel function targets were met. As this project began to show the results reported here, this particular surgeon began to change his practice to conform to the ERAS pathway and began to enjoy the same results. This is a testament to the effects of ongoing ERAS team meetings and review of outcome data as a method to promote ERAS.

The surgical practice in this study is a true “private practice,” whose physicians are not part of a greater health system and not affiliated with the anesthesiology group other than shared clinical work. In this environment, leadership from the department of anesthesiology was crucial in forming and sustaining the multidisciplinary workgroup, coordinating stakeholders from all phases of care, and aligning the goals of medical staff with those of the hospital. Taking a leadership role in value propositions in perioperative medicine, such as ERAS, can be an impactful way for private anesthesiology groups to demonstrate value to the facilities they serve.

Funding for an ERAS program coordinator and research nurse can be challenging to obtain in a community hospital, which can make auditing compliance for ERAS data points, as well as post-discharge surveillance, difficult. In fact, funding from the institution has proven to be among the most difficult parts of this project. However, the presentation of the results has made clear to the hospital that this is a program worth funding, and an ERAS coordinator position has been created and funded while this manuscript was in the making. It is the position of the authors that funding for the ERAS coordinator position should come from the hospital in this setting, as the hospital stands to recognize the cost savings associated with reduced length of stay.

Since this evaluation, we have expanded the anesthesia-led ERAS team model to colorectal surgery, ventral hernia repair, multilevel spine fusion, and hip preservation surgery. The next steps, as we expand the ERAS program at our hospital, include filling the ERAS program coordinator position, expanding acute pain services to add modalities such as postoperative ketamine and lidocaine infusions to current multimodal protocols, implementing ERAS protocols in obstetrics, open gynecological surgery, and vascular surgery.

This article was written following the standing committee on professional exchange (SCOPE) guidelines [11].

## Conclusions

This report demonstrates that it is feasible to create an ERAS program at a community hospital with mixed surgical staff. The most important elements contributing to the success of this program were the formation of a dedicated ERAS team, excellent patient and staff education, protocol-driven care pathways, accurate data management, and leadership from the department of anesthesiology.
